# Antitumoral Drug Potential of Tryptophan-Betaxanthin and Related Plant Betalains in the *Caenorhabditis elegans* Tumoral Model

**DOI:** 10.3390/antiox9080646

**Published:** 2020-07-22

**Authors:** Paula Henarejos-Escudero, Samanta Hernández-García, M. Alejandra Guerrero-Rubio, Francisco García-Carmona, Fernando Gandía-Herrero

**Affiliations:** Departamento de Bioquímica y Biología Molecular A, Unidad Docente de Biología, Facultad de Veterinaria, Regional Campus of International Excellence “Campus Mare Nostrum″, Universidad de Murcia, 30100 Murcia, Spain; paula.henarejos@um.es (P.H.-E.); samanta.hernandez@um.es (S.H.-G.); mariaalejandra.guerrero@um.es (M.A.G.-R.); gcarmona@um.es (F.G.-C.)

**Keywords:** Antitumoral, betalains, *C. elegans*, phytochemical, chemotherapy, tryptophan-betaxanthin

## Abstract

Betalains are plants pigments identified as potent antioxidant molecules, naturally present in foods like beetroot and prickly pears. Although activities described for betalain-containing formulations include cancer prevention and treatment, the use of extracts instead of purified pigments has avoided the investigation of the real chemopreventive and chemotherapeutic potential of these phytochemicals. Three betalain-rich extracts and six individual pure betalains were used in this work to characterize the activity and to explore possible molecular mechanisms. The animal model *Caenorhabditis elegans* (tumoral strain JK1466) was used to evaluate the effect of betalains as chemotherapeutics drugs. An objective evaluation method of tumor growth in *C. elegans* has been developed to assess the possible antitumoral activity of the different treatments. This protocol allowed a fast and reliable screening of possible antitumoral drugs. Among the betalains tested, tryptophan-betaxanthin reduced tumor size by 56.4% and prolonged the animal’s lifespan by 9.3%, indicating high effectiveness and low toxicity. Structure–activity relationships are considered. Assays with mutant strains of *C. elegans* showed that the mechanism underlying these effects was the modulation of the DAF-16 transcription factor and the insulin signaling pathway. Our results indicate that tryptophan-betaxanthin and related betalains are strong candidates as antitumoral molecules in cancer treatment.

## 1. Introduction

Cancer is a frequent and sometimes lethal disease. As the human population ages, cancer incidence rises, and with it the urgency to develop more potent and less toxic antineoplastic drugs. Plants are a source of phytochemicals, which may be effective molecules in chemoprevention and treatment strategies for improving patients’ health. Phytochemicals induce a set of biological responses in animal cells, including enzyme inhibition, antioxidant activity, and regulation of cellular signaling pathways [1]. Among phytochemicals with biological activities in relation to cancer disease, betalains are of great interest for their potential medical impact.

Betalains are water-soluble nitrogen plant pigments which are characteristic of plants of the Caryophyllales order. They are divided into two structural groups: the yellow betaxanthins and the violet betacyanins. Both groups share betalamic acid as the structural backbone. It is condensed with amines and amino acids in betaxanthins and with *cyclo*-DOPA (cyclodihydroxyphenylalanine) in betacyanins [2].

It is noteworthy that, even without purifying, some betalain-containing extracts from plants have been postulated in patented formulations (ES2320380, US2003036565) for the treatment of cancer, since betalains have demonstrated antioxidant, anti-inflammatory, hepatoprotective, anticancer, anti-diabetes, anti-lipidemic, antimicrobial, radio protective, and anti-proliferative activities [3]. However, most of these studies have been carried out with extracts rather than with pure betalains, and thus the nature of the bioactive molecule has not been unambiguously elucidated. In addition, the experiments with pure betalains have been restricted to two pigments: indicaxanthin and betanin [4,5].

In the present study, the anticancer activity of betalains has been investigated by using the in vivo model *Caenorhabditis elegans*. This little worm of 1 mm in length offers many advantages for a high throughput screening of molecules, since it has a short generation time and is easily maintained in large numbers. In the model animal *C. elegans* mutations in genes involved in cell cycle regulation can lead to cell hyperproliferation. Normally, these formed ectopic tissues are integrated in the respective organs and rarely form a tumor. In contrast, the uncontrolled growth of germline cells can cause classic tumors, because these cells are mitotically active and fail to differentiate into oocytes. The germline tumors can be formed either by germline intrinsic (cell autonomous) and somatically based (non-autonomous) mechanisms. Interestingly the germline cells are the sole source of stem cells in *C. elegans* during adulthood; moreover, the germline tissue is the only one that can undergo apoptosis. Thus the germline of *C. elegans* provides a powerful model to study processes related to cell carcinogenesis [6]. One of the mutant strains available with germline defects is the JK1466. This *C. elegans* strain displays a mutation of the *gld-1* tumor suppressor gene. In *gld-1(q485)* mutants, the germ cells fail to exit from mitosis and continue to proliferate throughout the gonad, forming a germline tumor that is lethal to the animal [7]. As reported before, worm GLD-1 protein is orthologous to the human QKI (KH domain with RNA binding capacity). In *C. elegans* it is involved in oocyte fate by the inhibition of mitotic proliferation and promoting the entry into meiotic prophase [8]. Knockdown of *gld-1* causes germ cells to ectopically transdifferentiate into various somatic cell types, forming human germ cell tumor-like teratomas [9,10]. In this study, an objective evaluation method of tumor growth in this strain is developed to assess the possible antitumoral activity of natural extracts and pure betalains in vivo. Three different betalain-containing natural extracts and six pure betalains are assayed separately.

## 2. Materials and Methods

### 2.1. Chemicals

Chemicals and reagents were purchased from Sigma (St. Louis, MO, USA). Solvents were from Merck Chemicals Ltd. (Dorset, England). HPLC-grade acetonitrile and methanol were purchased from Labscan Ltd. (Dublin, Ireland). Distilled water was purified using a Milli-Q system (Bedford, MA, USA) to obtain MQ water.

### 2.2. Extraction, Production and Purification of Betalains

The following betalains were used: betanin, phenylethylamine-betaxanthin, indicaxanthin, phenylalanine-betaxanthin, and tryptophan-betaxanthin. Red roots of *Beta vulgaris* were utilized to extract betanin. Phenylethylamine-betaxanthin, indicaxanthin, phenylalanine-betaxanthin, and tryptophan-betaxanthin were obtained as immonium condensation products of betalamic acid with the amines 2-phenylethylamine, L-proline, L-phenylalanine, and L-tryptophan, respectively. Biotechnological production was carried out in microbial factories following the Guerrero-Rubio et al. published method [11]. Briefly, betalamic acid was produced by a novel and efficient DODA enzyme and then condensed with amino acids and individual amines in bioreactors to obtain the derived betalains. An anionic exchange chromatography and a C-18 solid phase extraction were carried out to purify these betalains [12]. The experimental design scheme of the obtention of the betalains used in this study is shown in Supplementary Scheme SI.

### 2.3. Prickly Pears and Beetroot Extracts

Red and yellow prickly pear fruits (*Opuntia ficus-indica*) and beetroot were supplied from a local market. The vegetables were processed to remove the peel, chopped into small pieces, and finally maintained at −20 °C in containers. Ten grams of each prickly pear were weighed in 50 mL centrifuge tubes and crushed using a spatula to form a soft pulp. Seeds were removed. Ninety grams of beetroot were weighed and crushed using a food mixer Osterizer. Then, a cheesecloth was used to remove tissue debris from extracts and the seeds of the prickly pears. The extracts were then diluted with 10 mL and 90 mL of sterile M9 buffer respectively, centrifuged at 5000 ×g, filtered through a nylon cloth and finally through a 22 μm sterile filter. The extracts prepared were stored at −20 °C until use.

### 2.4. C. elegans Strains and Culture Conditions

The *C. elegans* wild-type strain N2, JK1466 *(gld-1(q485)/dpy-5(e61) unc-13(e51)*), TJ356 (*zIs356* [*daf-16p::daf-16a/b::GFP + rol-6(su1006*)]), TJ375 (*hsp-16.2/GFP*), and CF1038 (*daf-16(mu86)*) strains were obtained from the Caenorhabditis Genetic Center (CGC, St Paul, MN, USA), which is supported by the National Institutes of Health—Office of Research Infrastructure Programs (P40 OD010440). The strains were maintained at 20 °C in solid nematode growth medium (NGM) [13] and the experiments were performed in liquid S medium [13] with animals age-synchronized [13]. *Escherichia coli* OP50 was used as a food source to N2, TJ375, TJ356, and CF1038 strains and *E. coli* HT115 *gld-1* was used in order to feed JK1466 strain. *E. coli* was grown overnight in Luria-Bertani (LB) medium at 37 °C and was concentrated 10× in sterile M9 buffer.

### 2.5. Germline Tumor Growth Assays

The *gld-1*(q485) mutation causes a defect in oocyte development that results in growth of germline tumors that fill the somatic gonad, eventually leading to the animal’s death [7]. To examine the effects of the betalains and extracts on *gld-1*(q485) germline tumors, age-synchronized JK1466 worms were maintained on S medium with HT115 *gld-1* bacteria as a food source and the presence of the compound to assay. Worms were assayed at 20 °C with different concentrations of natural extracts at 0.05, 0.1, 1% *w/v* and the pure pigments in a final concentration of 25 μM. On the 4th day of adulthood, animals were washed with M9 buffer and mounted onto glass slides containing 10 mM sodium azide to anesthetize them. Bright-field (BF) images were taken using the 20× lens in a Leica DM 2500 LED microscope fitted with a Leica DFC550 camera (Leica Microsystems, Wetzlar, Germany). Analyses of the gonad size were performed with the ImageJ software.

### 2.6. Betalains and Natural Extracts Treatment in C. elegans

*C. elegans gld-1* (q485) mutants arrested larvae, L1, were collected and transferred to 25 mL sterile flasks containing 250 μL of an *E. coli* HT115 *gld-1* culture 10× concentrated in M9 buffer and 0.05, 0.1, 1 and 10% *w/v* in final concentration of each natural extract or 25 μM of each pure betalain individually (following Guerrero-Rubio et [14,15]) for a final volume of 5 mL. Sterile S medium was added and the flasks were kept under orbital shaking at 20 °C. Wild-type strain N2 and CF1038 strains were fed with *E. coli* OP50 [13]. Incubation time of these strains was 48 h for the survival assays [14] and on the 4th day of adulthood for tumor growth assays with the JK1466 strain. *C. elegans* strains treatment with betalains and subsequent assays are summarized in Supplementary Scheme SII.

### 2.7. Antioxidant Activity In Vitro by the ABTS Method

Betalains were assayed for antiradical capacity by following their effect on the free radical ABTS^•+^ (2,2’-azino-bis(3-ethylbenzothiazoline-6-sulfonic acid)). Decolorizing activity of the different betalains in various concentrations on ABTS^•+^ solutions was monitored at λ = 414 nm as previously described [15]. Trolox (6-hydroxy-2,5,7,8-tetramethylchroman-2-carboxylic acid) was used as a reference to normalize the results to TEAC (Trolox equivalent antioxidant activity) units.

### 2.8. Antioxidant Activity In Vivo

The capacity of the tested betalains to reduce oxidative stress in the animals was measured using the *C. elegans* mutant strain TJ375, following a previously published protocol [14].

### 2.9. Intracellular Localization of daf-16 Transcription Factor

*C. elegans* strain TJ356 (*daf-16*::*GFP*) expresses green fluorescent protein (GFP) fused with the transcription factor DAF-16, and determinate conditions induce the translocation of DAF-16 from the cytoplasm into the nucleus. TJ356 worms were synchronized with the normal procedure and then treated with 100 μM of the tested betalains or 1% of the natural extracts, in S medium supplemented with *E. coli* OP50 at 20 °C. After 72 h the worms were washed with M9 buffer and mounted onto glass slides containing 10 mM sodium azide as anesthetic. Images were taken at 20× magnification, using the GFP filter of the fluorescence microscope Leica DM 2500 LED microscope fitted with a Leica DFC550 camera (Leica Microsystems, Wetzlar, Germany). Worms were catalogued as nuclear if GFP was visible in the nuclei, cytoplasmic if GFP was diffused throughout the body of the worm, and intermediate if there were nuclei but there was some GFP fluorescence in the background. Positive control was prepared by “heat shocking” non-treated TJ356 worms at 37 °C for thirty minutes. Water was used as negative control.

### 2.10. C. elegans Tumor Induction via Gene Knockdown with RNAi

RNAi feeding was employed to ensure all worms had been silenced in *gld-1*. The JK1466 strain and wild-type strain used to propagate the tumoral phenotype had silenced *gld-1* gene. The *E. coli* strain HT115 (DE3) with the homologous DNA sequence for the *gld-1* (T23G11.3) gene inside the vector L4440 (pPD129.36) was obtained from Source BioScience (sourcebioscience.com) from the library*“ RNAi Library (Ahringer)”*. RNAi was performed by feeding according to standard protocols [16]. Briefly, the HT115 strain was cultured in LB supplemented with 30 µg/mL of carbenicillin overnight at 37 °C, the cells were then induced with 1 mM of IPTG (isopropyl β-D-1-thiogalactopyranoside) at 37 °C for one hour. Cultures were centrifuged to remove LB and concentrated to 10× with M9 buffer. Betalain treatment was done as described above including in the reaction medium 30 µg/mL of carbenicillin and IPTG to a final concentration of 1 mM.

### 2.11. Tumor Size Evaluation

An objective evaluation method of tumor size was needed to assess the possible antitumoral activity of natural extracts and pure betalains. Worms 20× BF images were used to outline and measure the area of each gonad by using the ImageJ software. Gonad sizes were measured from the loop region to the proximal region, including uterus area when it was filled with tumorous cells. In addition, to characterize gonad tumor morphology of the JK1466 strain, gonads were dissected and DAPI (4′,6-diamidino-2-phenylindole) -stained following the protocol by Barth Grant, adapted from R. Francis (Schedl Lab) and Sarah Crittenden (Kimble Lab) [7,17], with some modifications. Briefly, worms were transferred to depression slides and they were anesthetized with 10 mM sodium azide. A hypotonic medium at 0.7% M9 buffer was used to promote the complete extrusion of the gonads. Then, worms were decapitated using two 30-gauge syringe needles. Later, samples were stained with DAPI, as described below. Worms which were Acridine Orange stained followed the same gonad dissection protocol in order to characterize gonad tumor morphology.

### 2.12. DAPI and Acridine Orange Staining Processes

Animals from same assays and age were used to implement two different staining methods. For the DAPI (4′,6-diamidino-2-phenylindole) stain, dissected gonads were fixed and permeabilized with 100% methanol at −20 °C for 5 min, and then washed in PBT (PBS + 0.1% Tween 20) three times, and stained using 100 ng/mL DAPI in PBT [18]. Germ cell nuclei were then visualized as detailed below. For the Acridine Orange stain, whole worms were stained with acridine orange in 5 mL M9 buffer supplemented with 100 µL of OP50 and 25 µL from an acridine orange stock 10 mg/mL in MQ water [19]. Worms were left in an orbital shaker at 100 rpm for one hour at 20 °C. Later, the worms were washed with M9 buffer and left in 5 mL of M9 buffer for 3 h at 20 °C to assure complete washing. Images were collected using the 10×, 20×, and 40× lenses on a Leica DM 2500 LED microscope fitted with a Leica DFC550 camera (Leica Microsystems, Wetzlar, Germany) with the Leica I3 filtercube to visualize Acridine Orange stained worms, and with the Leica A filtercube to visualize DAPI stained samples.

### 2.13. Survival Assays

Animal lifespan was determined using the Lifespan machine, which is a platform for the automatic control of *C. elegans* mobility [20]. The mechanical device was built [14] to follow and analyze the *C. elegans* life cycle by taking images of the worms settled in analysis plates every hour and estimating the lifespan with a mathematical software. After 48 h in liquid media, the JK1466 worms were centrifuged at 2000× *g* and washed with M9 buffer three times. The procedure for survival assays was as previously described by Guerrero-Rubio et al. [14]. Briefly, 40–50 worms were then transferred to 35 mm petri dishes which contained 8 mL of NGM agar, 30 μg/mL of nystatin, 30 μg/mL of carbenicillin, 10 μg/mL of FUdR (2′-deoxy-5-fluorouridine), IPTG in a final concentration of 1 mM, and the tested compounds in the same concentration as in the liquid media. Plates were seeded with 100 μL of *E. coli* HT115 *gld-1* from an overnight culture in LB at 37 °C. After incubation for 20 min at 20 °C, closed lid plates were arranged into the lifespan machine. The experiments were performed in triplicate at 25 °C for 25 days. The same procedure was used for the CF1038 strain but using OP50 as a food source and avoiding IPTG.

### 2.14. Statistical Analysis

OASIS 2 was used to obtain the mathematical analysis of the lifespan data recorded [21], with the Kaplan–Meier estimator, Boschloo’s Test, Kolmogorov–Smirnov Test, and Survival Time F-Test.

Statistical analysis of the rest of the data was performed using the online tool Social Science statistics (https://www.socscistatistics.com) based in R. One-way ANOVA Calculator for independents measures was performed for numeric data, while the Chi-square Calculator Contingency Table was used for nominal data, and the significance level for all the data was 0.05.

## 3. Results and Discussion

### 3.1. Effects of Natural Extracts on Tumoral C. elegans Strain JK1466

The effects of three natural extracts were compared on tumor growth and survival rate of the mutant *C. elegans* strain JK1466. The betalain-containing natural extracts used were obtained from red and yellow *Opuntia ficus-indica* cactus pears and red roots of *Beta vulgaris*. Some studies have demonstrated that prickly pear and beetroot extracts are effective growth inhibitors and apoptosis inductors in several cancer cell lines [4,22,23]. In addition, the reduction of tumor formation in mice by oral administration of *B. vulgaris* extracts has been demonstrated, with a 60% reduction in the number of mice with adenomas in lung tumors and with an additional 30% reduction in the number of tumors for the affected animals [22]. Skin tumor formation induced chemically and promoted by ultraviolet (UV)-based light was also inhibited after the oral administration of *B. vulgaris* extracts in mice [24]. The tumor incidence in the liver was reduced by 40% [24] and chemoprevention against induced esophageal carcinogenesis was also demonstrated in rats for *B. vulgaris* extracts administered orally [25]. Results showed a reduction of 45% in the number of papillomas. Furthermore, aqueous extracts of *Opuntia ficus-indica* pears were able to inhibit tumor growth in a nude mouse ovarian cancer model compared with untreated animals [23]. *Opuntia* fruit extracts have also demonstrated their potential in the protection and recovery of the liver after damage has been induced [26].

**Figure 1:**
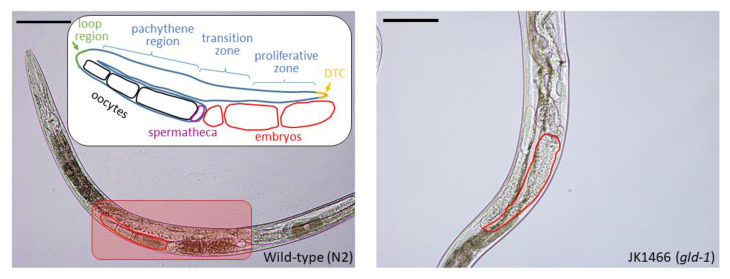
Objective evaluation method of tumor size in *Caenorhabditis elegans*. Comparison of germline phenotype of wild-type hermaphrodites and the *gld-1* mutants. Each panel shows one adult hermaphrodite on day 4 of adulthood. Gonad arms have been outlined in red and measured as viewed in BF (Bright-field) with 20× lens in a Leica DM 2500 LED microscope. Scale bar, 100 μm. On the left, BF image of a wild-type worm and their developed oocytes. Outlined region from loop region to proximal region has been measured (2100 μm^2^). Inset: Scheme for normal gonad morphology outlining zones and the development from oocytes to embryo. On the right, BF image of a *gld-1* mutant which contains proliferative cells throughout the entire gonad arm. Outlined region from loop region to proximal region has been measured (6836 μm^2^). Scale bar, 100 μm.

In *C. elegans gld-1*(q485) mutants, the germ cells fail to exit from mitosis and continue to proliferate throughout the gonad, forming a germline tumor (teratoma) that is lethal to the animal [7,9]. Teratomas originate from the dysregulation of cell pluripotency, as reported from different models in vivo (*C. elegans*, mouse, planaria) and in vitro (embryonic stem cells, epiblast stem cells). While the underlying mechanism of teratoma formation remains unclear the RNA-binding proteins as GLD-1 in worms or DND1 in murine had a key role in it [27]. In humans ovarian teratoma (ovarian germ cell tumors, GCTs) originate from primordial germline cells and are approximately 30% of all ovarian neoplasms, and while most of them are benign they need to be treated to avoid malignant transformation in squamous cell carcinoma or in adenocarcinoma which in most of the cases have poor prognosis for the patients [28]. Thus, *gld-1*(q485) mutants are a model for germline teratoma formation and development. In wild-type worms, cells move from the distal end to the loop region going through the proliferative, transition zone, and pachytene region. Gamete formation begins after the loop region, where oocytes are developed (Figure 1). *gld-1*(q485) mutants contain proliferative cells throughout the entire gonad arm instead. To examine the effects of natural extracts on tumor growth, gonad sizes were measured from loop region to the proximal region, including the uterus area when it was full of tumorous cells. This area was selected since it is swelled and enlarged by tumor development [6], as shown in Figure 1.

**Figure 2:**
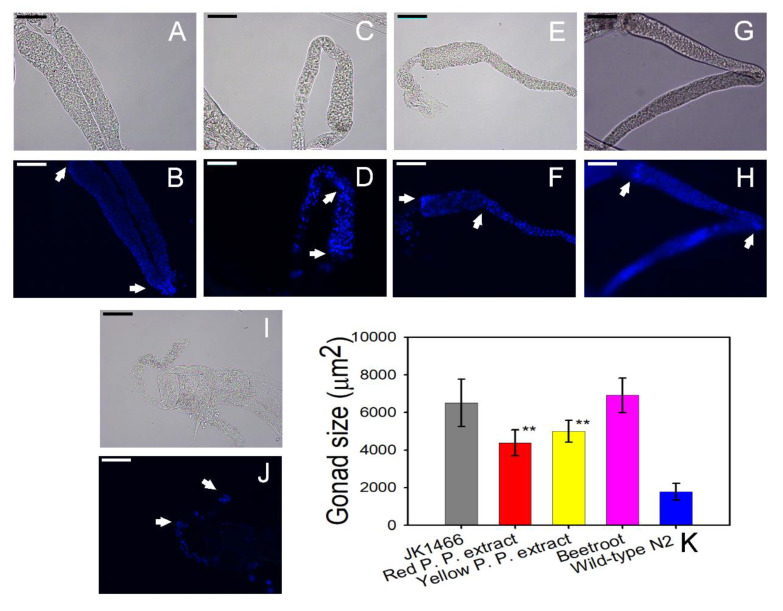
Size and morphology comparison of *gld-1* mutants’ gonads with and without treatment with betalain-containing extracts and the wild-type animal. Representative images of BF and DAPI-stained of dissected gonads from *glp-1*(q485) animals treated with natural extracts (at 1% *w/v*) and wild-type animals are shown. Scale bar, 50 μm. Dissected gonads images from *gld-1* mutants without treatment (positive control) in BF (**A**) and DAPI-stained (**B**). Dissected gonads images from *gld-1* mutants treated with red prickly pear extract in BF (**C**) and DAPI-stained (**D**), treated with yellow prickly pear extract in BF (**E**) and with DAPI-stained (**F**), and treated with beetroot extract in BF (**G**) and DAPI-stained (**H**). Dissected gonads images from wild-type N2 strain (negative control) are shown in BF (**I**) and DAPI-stained (**J**). Gonads from loop region to proximal region have been indicated with arrow marks. (**K**) Gonads size histograms (mean ± SEM) of wild-type (negative control) (*n* = 17) and *gld-1* mutants treated with red (n = 13) and yellow (*n* = 13) prickly pears extracts and beetroot extract (*n* = 18) and without treatment (positive control) (*n* = 18). ** indicated *p* ≤ 0.05.

In a preliminary screening assay, natural extracts with different concentrations 0.05%, 0.1%, 1%, and 10% *w/v* were assayed to identify the effective dose. At 10%, the measurements were found impossible to execute due to large number of deaths. Among the remaining three concentrations, 1% (*w/v*) showed the highest tumor gonad reduction. The greatest effect on tumor reduction was obtained for red prickly pear extracts, which reduced the size of tumors by 45% taking the size of the wild-type gonad as the normal reference. The yellow prickly pear extract reduced tumor size by 31.9% (Figure 2K). Beetroot extract did not have a significant effect on the gonad tumor size of *C. elegans* (Figure 2K). As shown in Figure 2, gonads from *gld-1* mutants without treatment (Figure 2A,B) and gonads from *gld-1* mutants which were treated with beetroot extracts (Figure 2G,H) presented tumors of larger sizes and with a higher number of cell nuclei, which were visualized after DAPI staining. However, gonads from *gld-1* mutants which were treated with red (Figure 2C,D) and yellow (Figure 2E,F) prickly pear extracts showed smaller sizes and fewer cell nuclei in the DAPI stain. Figure 2I,J shows the size and the number of cell nuclei of a gonad from wild-type *C. elegans* used as controls. As it can be appreciated, the gonad size is smaller and has less cell nuclei than the tumor-induced specimens.

These data demonstrate that worms that were fed with prickly pear extracts had less cell proliferation in vivo, and that the gonad tumor sizes were significantly reduced in respect to controls.

### 3.2. Natural Extracts Effect on the Lifespan of the Tumoral Worms

**Figure 3:**
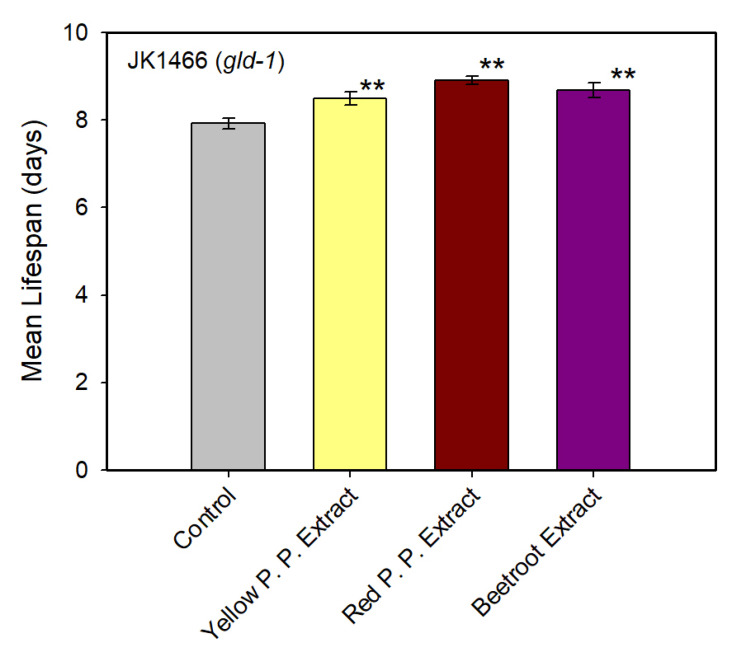
Mean lifespan of JK1466 mutant worms treated with natural extracts at 1% *w/v*. Mean lifespan (mean ± SE) is shown for *C. elegans gld-1* mutants which were treated with yellow (*n* = 60) and red (*n* = 46) prickly pear extracts and beetroot extract (*n* = 65), and without treatment (control) (*n* = 48). ** indicated *p* ≤ 0.05.

After determining the effective concentration for tumor gonad reduction, survival assays were made with natural extracts at 1% *w/v*. The analysis performed in the Lifespan Machine shows a significant increase in the average lifespan of *C. elegans* in the presence of 1% *w/v* from red prickly pear extract, where the mean survival increased from 7.9 to 8.9 days (Figure 3). Therefore, the mean survival increased by 12.5%. The beetroot extract at 1% also increased the mean survival from 7.9 to 8.7 days (Figure 3), with a mean survival increase of 9.6%. The yellow prickly pear extract at 1% increased the mean survival of the worms from 7.9 to 8.5 days (Figure 3), with a mean survival increase of 7.3%. All mean survival increases recorded were statistically significant (*p* < 0.05). Therefore, red prickly pear was not only able to efficiently reduce the tumor growth but also to increase the lifespan of JK1466 *C. elegans* in a high extent. Wild-type *C. elegans* N2 strain showed a longer lifespan after feeding with yellow prickly pear extract than with the red prickly pear extract. Also, the effective concentration was different in this strain in comparison to the JK1466 strain [14]. This may indicate a different mode of action between the two extracts, with the red one being more effective in the reduction of tumor size and extension of lifespan under these conditions.

Beetroot extracts did not have a significant effect on tumor growth in *C. elegans* whereas in others studies with mice it had a great effect [3,22,24,25]. In our study, the coloration of the beetroot extract and its appearance during the assay changed. It showed a brown color and aggregates which may correspond to oxidation products [29]. S medium contains different metals, which may react with the different compounds of the beetroot extract. This would reduce the stability and bioavailability of betanin in the extracts and reduce a possible positive effect in the nematode [30]. Betanin is the main pigment present in red beetroot [31], and thus pure betanin was assayed in a further experiment. In addition, betanin is the main pigment present in red fruits of *Opuntia ficus-indica*, while indicaxanthin is the main pigment present in yellow fruits of the same species [32] as showed the HPLC chromatograms of the used extracts (Supplementary Figure S1). These and other different betalains were obtained and purified in order to assay pure individual molecules in tumor reduction and lifespan experiments.

### 3.3. Effect of Pure Betalains in Tumoral C. elegans Strain JK1466

**Figure 4:**
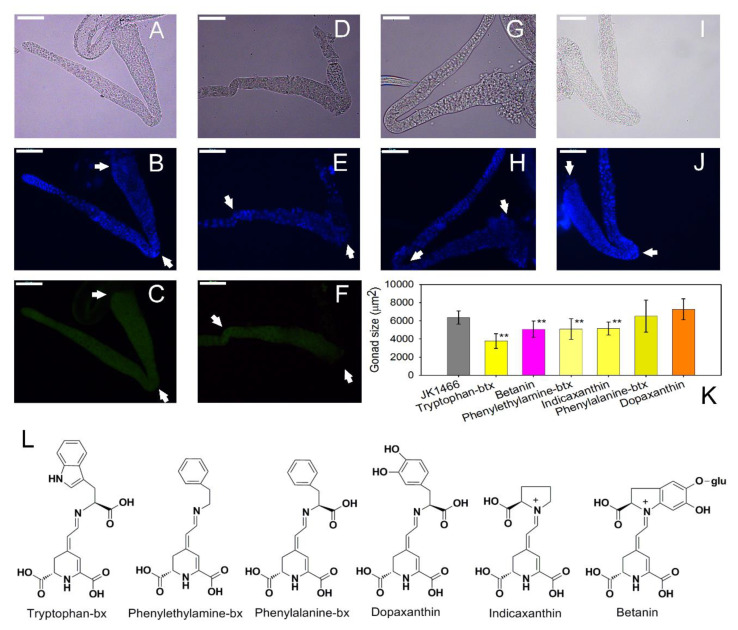
Size and morphology comparison of the gonads of *C. elegans gld-1* mutants with and without treatment with pure betalains (25 μM). Representative images of BF, DAPI-stained and Acridine Orange-stained of dissected gonads from *glp-1*(q485) animals treated with pure betalains and without treatment. Scale bar, 50 μm. Dissected gonads images from *gld-1* mutants without treatment (positive control) in bright field (**A**), DAPI (**B**), and Acridine Orange-stained (**C**). Dissected gonads images from *gld-1* mutants treated with tryptophan-betaxanthin in BF (**D**), DAPI (**E**), and Acridine Orange-stained (**F**). Dissected gonads images from *gld-1* mutants treated with betanin in BF (**G**) and DAPI-stained (**H**), and treated with phenylethylamine-betaxanthin in BF (**I**) and DAPI-stained (**J**). Gonads from loop region to proximal region have been indicated with arrow marks. (**K**) Gonads sizes (mean ± SEM) histograms of *gld-1* mutants treated with the pure betalains tryptophan-betaxanthin (*n* = 13), betanin (*n* = 8), phenylethylamine-betaxanthin (*n* = 17), indicaxanthin (*n* = 15), phenylalanine-betaxanthin (*n* = 14), dopaxanthin (*n* = 12) and without treatment (positive control) (*n* = 15). Structures for all the pure betalains studied in this work are shown (**L**). glu: glucose; bx: betaxanthin.** indicated *p* ≤ 0.05.

Six pure betalains (Figure 4L) were compared for their effects on the tumor growth of mutant JK1466 *C. elegans*. This collection included the yellow betalains phenylethylamine-betaxanthin, tryptophan-betaxanthin, indicaxanthin, dopaxanthin, and phenylalamine-betaxanthin and the violet betalain betanin. Phenylethylamine-betaxanthin, indicaxanthin, phenylalamine-betaxanthin, and betanin are present in *Opuntia ficus-indica* [2]. Betanin and phenylalamine-betaxanthin are present in *Beta vulgaris* [2,31]. Dopaxanthin is a pivotal pigment of the biosynthetic pathway of betalains and it has the highest antioxidant activity in vitro [2]. Tryptophan-betaxanthin is a compound found in plants of the traditional Chinese medicine (TCM). This betalain has been investigated for its potential binding affinity to sirtuin Sirt1 protein using different computational scoring methods in relation to diseases associated with aging [33].

In order to examine the effect of pure betalains on tumor growth, gonad sizes were measured from the loop region to the proximal region, including uterus area when it was filled of tumorous cells. Treatments with 25 μM of each pure betalain were assayed individually. None of the pigments showed a negative response in tumor size. The greatest effect on tumor reduction was caused by tryptophan-betaxanthin, which reduced the tumor size by 56.4% (Figure 4D–K), followed by pure betanin, phenylethylamine-betaxanthin and indicaxanthin, which reduced tumor sizes by 28.0, 27.7 and 26.3% respectively (Figure 4K). Phenylalanine-betaxanthin and dopaxanthin did not have a significant effect on the tumor of *C. elegans* (Figure 4K). These data demonstrate that worms which were fed individually with pure betanin, phenylethylamine-betaxanthin, indicaxanthin, and above all with tryptophan-betaxanthin reduced tumor growth significantly.

Betalains were confirmed to remain in the medium during the in vivo assays. Betanin experienced degradation but 25% of the initial pigment remained after 48 h and betaxanthins were more stable with 75–100% of the initial pigment remaining after 48 h at 20 °C in the dark (Supplementary Figure S2). All betalains are known to possess high antioxidant and antiradical activities. However, dopaxanthin - the betalain with the highest antioxidant capacity did not have a significant effect on the tumor growth of *C. elegans*. These results show that there is not a direct link between the antioxidant capacity of the pigments and their antitumoral effect. In the present study tryptophan-betaxanthin has been identified as the most effective pure betalain in tumor size reduction in *C. elegans*. Tryptophan-betaxanthin, besides its antioxidant and antiradical activities, has been identified as a potential inhibitor of Sirt1 (silent information regulator 1) protein by in silico studies [33]. Our results are the first experimental evidence of a health-promoting effect of this molecule, which could be a possible candidate in novel treatment strategies towards diseases as cancer.

### 3.4. Survival Assays in the Tumoral Strain JK1466

**Figure 5:**
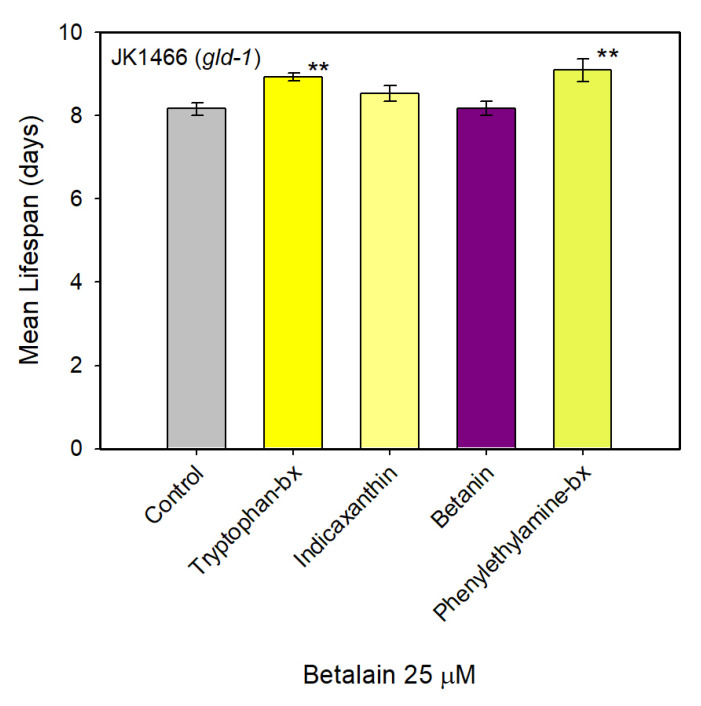
Mean lifespan of *C. elegans* JK1466 strain treated with pure betalains. Mean lifespan (mean ± SE) of *C. elegans gld-1* mutants which were treated with tryptophan-betaxanthin (*n* = 62), indicaxanthin (*n* = 60), betanin (*n* = 52), and phenylethylamine-betaxanthin (*n* = 50) and without treatment (control) (*n* = 64). ** indicated *p* ≤ 0.05.

Among the pure betalains assayed, those with the highest inhibition of tumor growth were selected for performing survival assays. Thus, experiments were made with betanin, phenylethylamine-betaxanthin, indicaxanthin and tryptophan-betaxanthin in *C. elegans* JK1466 strain. The analyses of lifespan were performed in the automatic platform based on the Lifespan Machine. Results show a significant increase in the average lifespan of *C. elegans* in the presence of phenylethylamine-betaxanthin, where the mean survival time increased from 8.2 to 9.1 days (Figure 5). Therefore, the mean survival time increased by 11.4%. Tryptophan-betaxanthin also increased the mean survival by 9.3%, from 8.2 to 8.9 days (Figure 5). All mean survival increases determined are statistically significant (*p* < 0.05). Indicaxanthin and betanin pigments did not have a significant effect on the lifespan of *gld-1* mutants. Therefore, phenylethylamine-betaxanthin and tryptophan-betaxanthin not only reduced the tumor growth but also increased the lifespan of JK1466 *C. elegans* significantly.

### 3.5. Antioxidant Activity

**Figure 6:**
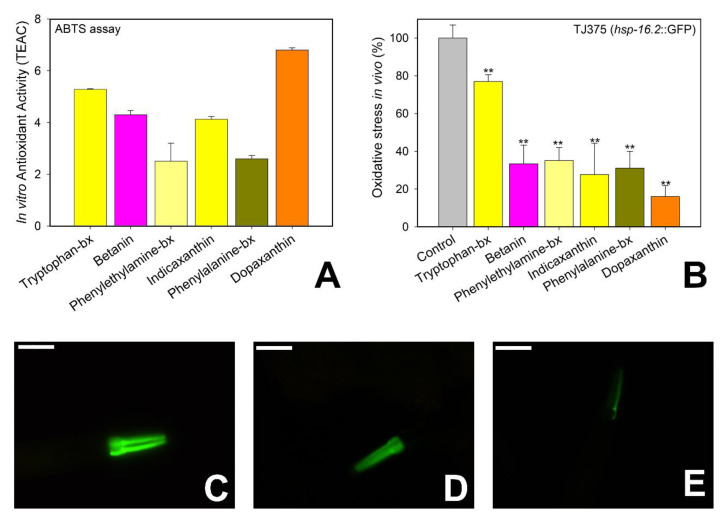
Antioxidant effects of the pure betalains tested in vitro and in vivo. (**A**) Results of the antioxidant capacity in vitro measured by the ABTS assay. (**B**) Results of the antioxidant activity in vivo with the *C. elegans* strain TJ375 treated with the tested betalains (25 μM) and exposed to the oxidant agent juglone (20 μM). (**C**–**E**) Representative fluorescence images of the strain TJ375, (**C**) control animal exposed to juglone, (**D**) animal treated with tryptophan-betaxanthin (25 μM), (**E**) animal treated with dopaxanthin (25 μM). ** Statistically significant at *p* ≤ 0.05. Scale bar: 50 μm.

The antioxidant capacity of the tested natural molecules is shown in Figure 6. First, the ABTS assay was performed to evaluate their antioxidant activity in vitro (Figure 6A) using TEAC units (Trolox equivalent antioxidant activity), followed by an in vivo assay (Figure 6B–E) using the *C. elegans* mutant strain TJ375. This strain has the heat shock protein HSP-16.2 fused with GFP, and the expression of this protein is inducible by heat or an oxidant molecule such as juglone. When the animals were exposed to 20 μM of juglone the fused protein HSP-16.2::GFP accumulated in the pharynx of the animals producing a bright green fluorescence as shown in Figure 6C (control) that can be measured with image analysis software. Treatment with pure betalains reduced the expression of the protein (Figure 6B,D,E) when compared with non-treated controls (Figure 6B,C), with dopaxanthin being the most effective antioxidant molecule in vitro and in vivo (Figure 6E).

The antioxidant capacity of betalains and betalain-containing natural extracts is well known [34,35,36] and it is modulated by each individual substructure [37]. Structurally all betalains contain a betalamic acid moiety that can be considered the bioactive unit. It has an antioxidant capacity of 2.7 TEAC units as reported by Gandia-Herrero and coauthors [38]. The antioxidant activity assay performed in vitro for the tested betalains in this work using Trolox as a control confirmed dopaxanthin as the most antioxidant pigment (6.8 TEAC units) (Figure 6A). It was followed by tryptophan-betaxanthin (5.28 TEAC units) and betanin (4.3 TEAC units). On the other hand, phenylethylamine-betaxanthin (2.7 TEAC units) and phenylalanine-betaxanthin (2.6 TEAC units) showed similar antioxidant activity to betalamic acid. These results indicate that the antioxidant capacity in vitro is indeed related to the structure and that it is increased by the presence of hydroxyl groups as well as by the presence of an indole group.

Dopaxanthin was also the most active antioxidant pigment in vivo, being able to reduce the oxidative stress of the worms by 84.0% (Figure 6B), followed by indicaxanthin (72.3%) and betanin (66.7%). Tryptophan-betaxanthin was the less antioxidant pigment assayed in vivo; nevertheless, the molecule was able to reduce the oxidative stress of the animals by 23.2%. The discrepancy between the in vitro and in vivo assays can be a consequence of the stability and the bioavailability of the molecules.

Several studies have reported the effect of antioxidant molecules against cancer in in vitro assays but also in preclinical studies [2,39], since they are able to neutralize reactive oxygen species (ROS) that otherwise may damage DNA and therefore start oncogenesis. In addition, several clinical trials have shown that the use of antioxidant supplements as adjuvant therapies to chemotherapy or radiotherapy may ameliorate the adverse effects of these treatments. CoQ10 supplementation decreases hair loss and prevents cardiotoxicity in patients treated with doxorubicin. Neurotoxicity produced by cisplatin treatment was reduced in patient with solid malignant tumors (lung, ovary, or testicle) by vitamin D supplementation [40]. However, vitamin C in high doses failed to improve either the symptoms or the survival of patients with an advanced stage cancer [41]. Moreover, recent studies have linked the proliferation of some cancers to the effects of antioxidants. The studies showed that the p53 factor induces an increase of ROS that activate several protection mechanisms including the cellular death by apoptosis. Thus, the presence of antioxidants may hinder the activation of cellular death, promoting cell proliferation [42,43,44]. The disparity of results suggests that there is no direct relationship between the antioxidant capacity and the antitumoral effect of one molecule as there are more factors involved as the cancer type, the molecule structure, and the underlaying mechanisms. Our results show that most antioxidant molecules both in vivo and in vitro were unable to reduce tumoral growth in the *C. elegans* model unlike tryptophan-betaxanthin that had little effect as an antioxidant in vivo o but reduced the tumor growth by 57% and increased the lifespan of the animals. The rest of the tested molecules showed more antioxidant activity than tryptophan-betaxanthin but were less effective at reducing the tumor. Altogether the results suggest that the positive effect of some of the tested betalains in tumoral growth and lifespan extension may be partially explained by their antioxidant capacity but not totally as there is no direct relationship between the antioxidant activity and the antitumoral effect. Nevertheless, targeting ROS with antioxidants is a good approach to cancer therapy, since mitochondrial redox signaling activates protection mechanisms such as HIF and AMPK which improve cancer cell survival, as tumoral cells have higher levels of ROS than normal cells [45], produced by an abnormal cell proliferation, increased metabolism, and mitochondrial malfunction [46]. Thus, small antioxidant molecules able to inhibit ROS signaling may render cancer cells more vulnerable to ROS-generating chemotherapeutic agents [47].

### 3.6. Pure betalains and Natural Extracts Evaluation as Modulators of the Longevity Pathway

**Figure 7:**
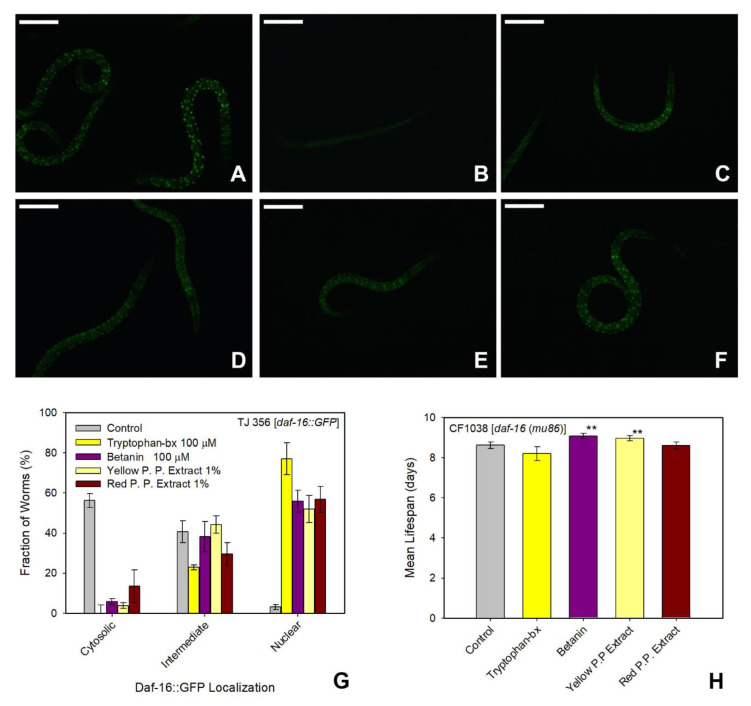
Effect of extracts and pure betalains on the longevity pathway. Representative fluorescence images from TJ356 *[daf-16::GFP]* strain, positive control (**A**), negative control, (**B**), treated with tryptophan-betaxanthin, (**C**), treated with betanin, (**D**), treated with yellow (**E**) and red (**F**) prickly pear extracts. Scale bar: 100 µm. (**G**) Histograms for the fraction of cytosolic, intermediate, and nuclear classification of worms of the TJ356 *[daf-16::GFP]* strain treated with tryptophan-betaxanthin (*n* = 48), betanin (*n* = 34), yellow (*n* = 52), and red (*n* = 44) prickly pear extracts and without treatment (*n* = 32). Two independent trials were measured. (**H**) Mean lifespan (mean ± SE) of *C. elegans [daf-16(mu86)]* mutant animals (CF1038 strain) treated with tryptophan-betaxanthin (*n* = 53), betanin (*n* = 40), yellow (*n* = 61), and red (*n* = 58) prickly pear extracts and without treatment (*n* = 62). ** indicated *p* ≤ 0.05.

Usually antioxidant molecules protect cells and organisms against oxidative stress either by radical scavenging or by modulation of transcription pathways like the insulin signaling pathways or the redox-active Nrf2/ARE signaling pathway [48]. To analyze the effects of the pure betalains and natural extracts tested in this work as modulators of the insulin signaling pathway, the *C. elegans* mutant strain TJ356 was used. The TJ356 strain expresses GFP fused with the DAF-16 transcription factor, homologue to the mammalian transcription factor FOXO. When the insulin signaling pathway is inactivated the GFP fluorescence is translocated from the cytoplasm to the nuclei of the cells. As shown in Figure 7C,G, tryptophan-betaxanthin induced a 77.1% nuclear translocation of DAF-16, an indication that the molecule is able to activate the insulin signaling pathway. This result supports the findings in Kuan-Chung et al. [33], where a modulation of SIR-2.1 (mammalian Sirt 1 protein) could activate DAF-16 transcription factor which ultimately activates the target gene superoxide dismutase (*sod-3*) [49], resulting in protection against oxidative stress and therefore in a lifespan extension [50]. In the same way betanin and the prickly pear extracts translocated the transcription factor DAF-16 from the cytoplasm to the nuclei, but the effect was less pronounced.

As discussed above, the pure betalains and natural extracts tested in this work expand the mean lifespan of the *C. elegans gld-1* mutants (JK 1466) and wild-type animals (N2 strain), with tryptophan-betaxanthin, betanin and the prickly pear extract being the most effective. However, the lifespan prolongation effects of tryptophan-betaxanthin and red prickly pear extract were completely lost in the *daf-16(mu86)* mutant animals (CF1038 strain), as shown in Figure 7H, Supplementary Figure S3, and Supplementary Table S1. This loss of effect suggests that the transcription factor DAF-16 is responsible for the lifespan extension in *gld-1* mutants and in wild-type worms treated with tryptophan-betaxanthin. The activation of the DAF-16 transcription factor could also explain the higher reduction of tumor size in the *gld-1* mutants treated with tryptophan-betaxanthin and red prickly pear extracts. Therefore DAF-16 transcription factor seems to be the mechanistic connection between tryptophan-betaxanthin treatment and the observed tumor reduction in the mutant phenotype. As Pinkston et al. [51] reported, the long-lived *C. elegans daf-2* mutants required DAF-16 to promote the germ line apoptosis, reduction in the number of germ cells and a lifespan extension in *gld-1; daf-2* mutants. Normally, tumor rates invariably rise as the animal ages. However, DAF-16 target genes that affect tumor growth do not affect aging, indicating that DAF-16 can regulate tumor growth and lifespan independently. Therefore, an activation of DAF-16 could increase the regulation of DAF-16 target genes involved directly or indirectly in cell proliferation and apoptosis. Thus, DAF-16 activity counteracts both aging and tumor growth in *C. elegans* [52].

The lifespan extension effect in *daf-16 (mu86)* mutants treated with yellow prickly pear extract and betanin was not completely lost, even though, as shown before, there was a translocation of the transcription factor DAF-16. The small lifespan extension shown in the assay was, most probably, due to a radical scavenging effect and protection against oxidative stress via heat shock proteins (HSPs) [14]. It was demonstrated that betanin and indicaxanthin (the main pigment present in yellow prickly pears) decreased the oxidative *stress in vivo* by 75% and 94% respectively [14]. 

## 4. Conclusions

Pure individual betalains and natural extracts have been proven to reduce tumor growth in vivo in the animal model *C. elegans*. The most effective molecule was tryptophan-betaxanthin which showed the highest effect on tumor size. Tryptophan-betaxanthin is a compound present in plants of TCM and its effect may be related to its being an inhibitor of Sirt1. The tested individual betalains and natural extracts reduced the tumor growth and expanded the lifespan of *gld-1* mutant animals. They act not only as antioxidants but also by regulating the DAF-16 transcription factor as one of the major players involved in the longevity pathway. With the results of the present work, betalains are revealed as potential anticancer phytochemicals, of possible importance in chemoprevention and treatment strategies.

## 5. Patents

There is a Spanish national patent in progress, partially resulting from the work reported in this manuscript. The title is: “Betaxantinas derivadas de triptófano y feniletilamina para uso en el tratamiento y/o prevención del cáncer” and the aplication number is P201930649.

## Supplementary Materials

The following are available online at https://www.mdpi.com/2076-3921/9/8/646/s1, Supplementary Scheme SI. Betalains obtention workflow. Betaxanthins were produced biotechnologically using a DODA enzyme expressed in an *E. coli* host. Betanin was obtained by extraction from *Beta vulgaris* roots, Supplementary Scheme SII. Experimental design scheme for different *C. elegans* strains treatment with betalains and subsequent assays, Supplementary Table S1: Lifespan assays survival data, Supplementary Figure S1. HPLC analyses of the extracts employed. HPLC chromatograms for: (A) beetroot extract, (B) red prickly pear extract, (C) pure betanin used as control, (D) yellow prickly pear extract and (E) pure indicaxanthin used as control, Supplementary Figure S2. Betalains stability in *C. elegans* S. medium after 48 h at 20 °C in the dark. The betalain content in the medium was obtained by measuring its absorbance spectrophotometrically at 536 nm for betanin and 480 nm for the betaxanthins (tryptophan-bx, phenylalanine-bx, dopaxanthin and indicaxanthin) at t = 0 and t = 48 h, Supplementary Figure S3. Survival plots for *C. elegans gld-1* mutants treated with betalain-containing natural extracts and pure betalains. Natural extracts (A). Pure betalains (B). Survival plots for *C. elegans* CF 1038 *[daf-16(mu86)]* treated with Tryptophan-betaxanthin, betanin and yellow and red prickly pears extracts (C).
