# Supplementary material for: Antitumoral Drug Potential of Tryptophan-Betaxanthin and Related Plant Betalains in the Caenorhabditis elegans Tumoral Model
Source: Antioxidants (Basel). 2020 Jul 22;9(8):646. doi: 10.3390/antiox9080646 (PMC7465535; doi:10.3390/antiox9080646)
Supplement: Supplementary file 1 [file antioxidants-09-00646-s001.pdf]

Supplementary Material

Antitumoral drug potential of tryptophan-betaxanthin and related plant betalains in the *Caenorhabditis elegans* tumoral model

Paula Henarejos-Escudero[1], Samanta Hernández-García[1], M. Alejandra Guerrero-Rubio[1], Francisco García-Carmona[1], Fernando Gandía-Herrero[1,*]

[1] Departamento de Bioquímica y Biología Molecular A, Unidad Docente de Biología, Facultad de Veterinaria. Regional Campus of International Excellence "Campus Mare Nostrum". Universidad de Murcia, 30100 Murcia, Spain; paula.henarejos@um.es (H.-E. P.); samanta.hernandez@um.es (H.-G. S.); mariaalejandra.guerrero@um.es (G.-R. M.A.); gcarmona@um.es (G.-C. F.); fgandia@um.es (G.-H. F.)

* Correspondence: fgandia@um.es; Tel.: (+34 868 889592); Fax: +34 868 884147

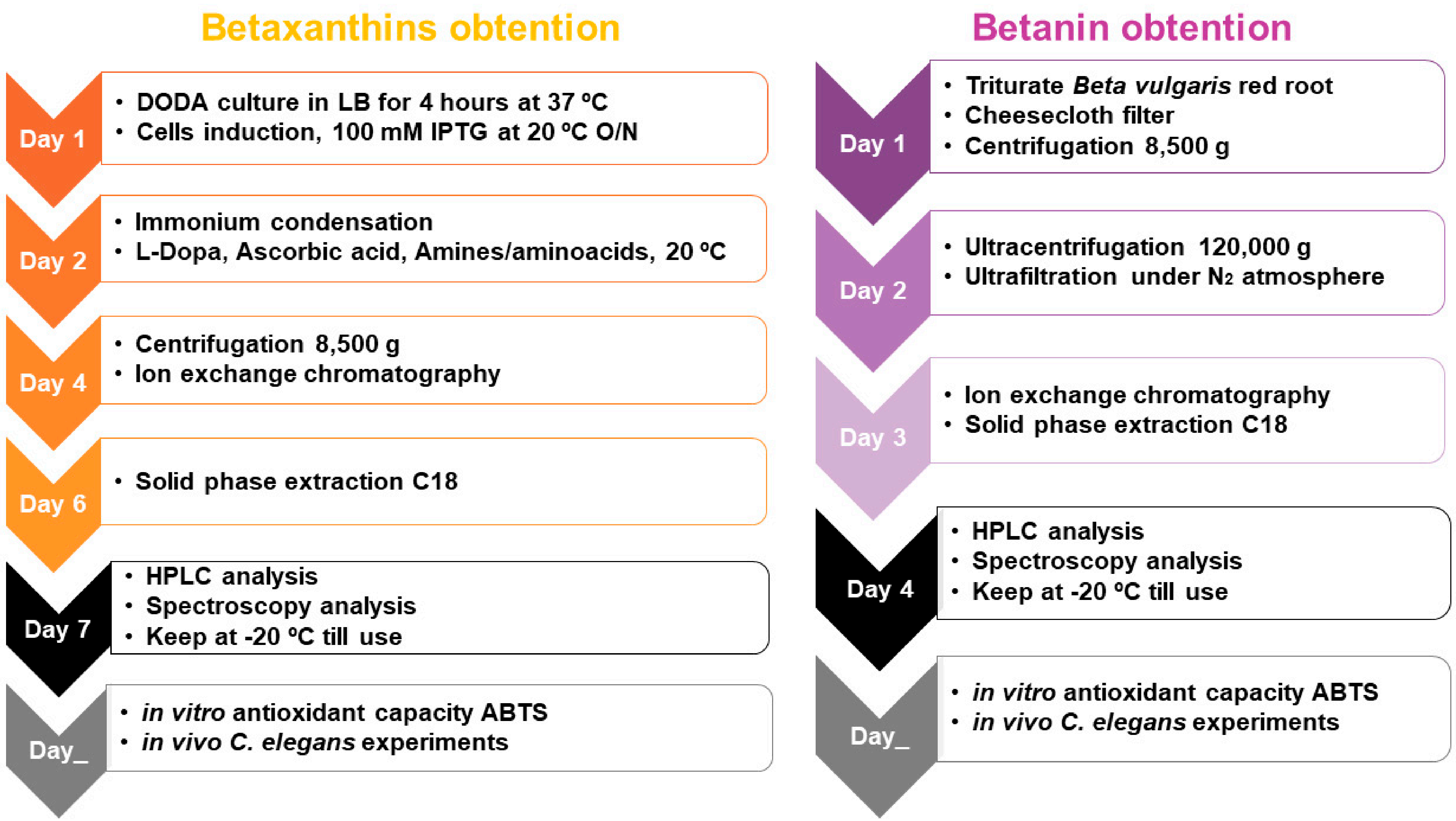

Supplementary Scheme SI. Betalains obtention workflow. Betaxanthins were produced biotechnologically using a DODA enzyme expressed in an *E. coli* host. Betanin was obtained by extraction from *Beta vulgaris* roots.

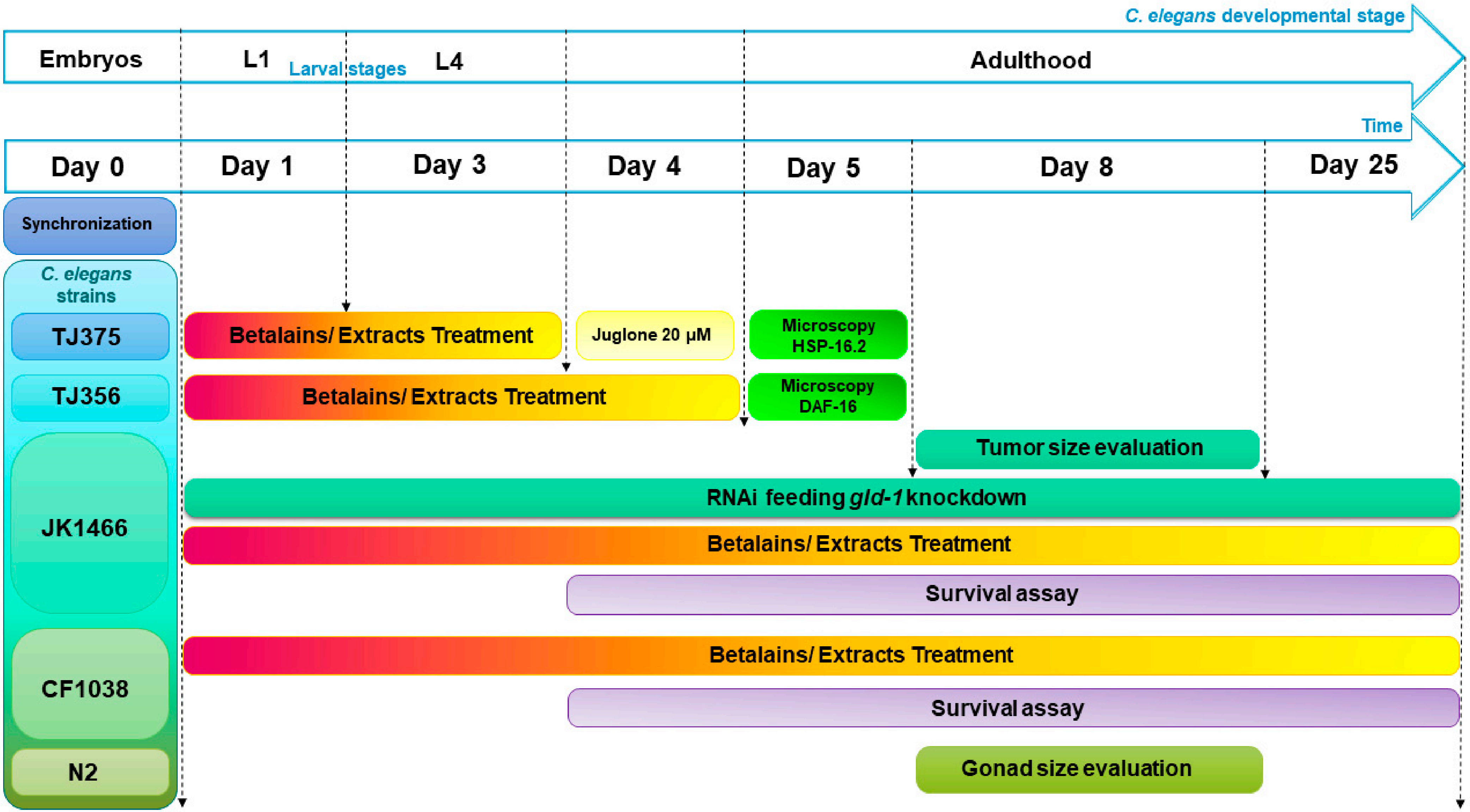

Supplementary Scheme SII. Experimental design scheme for different *C. elegans* strains treatment with betalains and subsequent assays.

Supplementary Table 1: Lifespan assays survival data

Strain	Treatment	nº	Mean Lifespan (days)	S. E	95 ci	Lifespan Change (%)	*p* value *vs* Control
**JK1466 [gld-1(-)]**	Control	48	7.92	0.12	7.68 ~ 8.16	0	
	Yellow P. P. Extract (1%)	60	8.5	0.15	8.21 ~ 8.79	7.32	2.30E-06
	Red P. P. Extract (1%)	46	8.91	0.09	8.73 ~ 9.09	12.5	4.10E-09
	Beetroot Extract (1%)	65	8.68	0.17	8.34 ~ 9.01	9.59	0.0103
	Control	64	8.16	0.15	7.86 ~ 8.47	0	
	Tryptophan-bx (25 µM)	62	8.92	0.09	8.74 ~ 9.11	9.59	0.0019
	Indicaxanthin (25 µM)	60	8.52	0.19	8.15 ~ 8.90	4.54	0.1356
	Betanin (25 µM)	52	8.17	0.17	7.83 ~ 8.50	0.12	0.9899
	Phenylethylamine-bx (25 µM)	50	9.09	0.27	8.55 ~ 9.63	11.74	0.0001
	Control	62	8.62	0.16	8.31 ~ 8.94	0	
	Tryptophan-bx (25 µM)	53	8.2	0.34	7.54 ~ 8.86	-4.87	0.3111
	Betanin (25 µM)	40	9.07	0.13	8.82 ~ 9.32	5.22	0.0313
	Yellow P. P. Extract (1%)	61	8.96	0.13	8.71 ~ 9.21	3.94	0.0165
	Red P. P. Extract (1%)	58	8.6	0.17	8.26 ~ 8.94	-0.23	0.2368

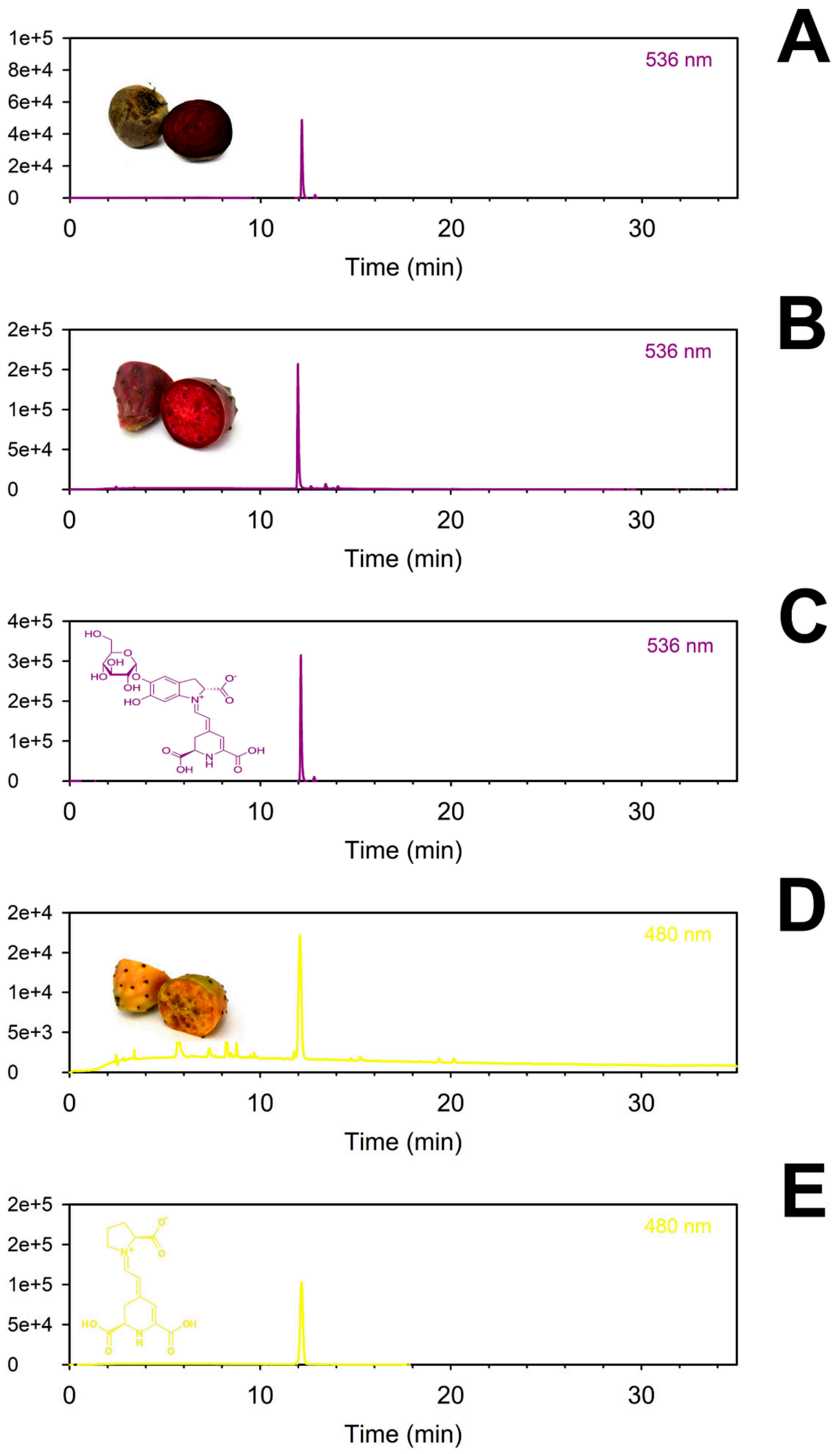

Supplementary Figure S1. HPLC analyses of the extracts employed. HPLC chromatograms for: (**A**) beetroot extract, (**B**) red prickly pear extract, (**C**) pure betanin used as control, (**D**) yellow prickly pear extract and (**E**) pure indicaxanthin used as control.

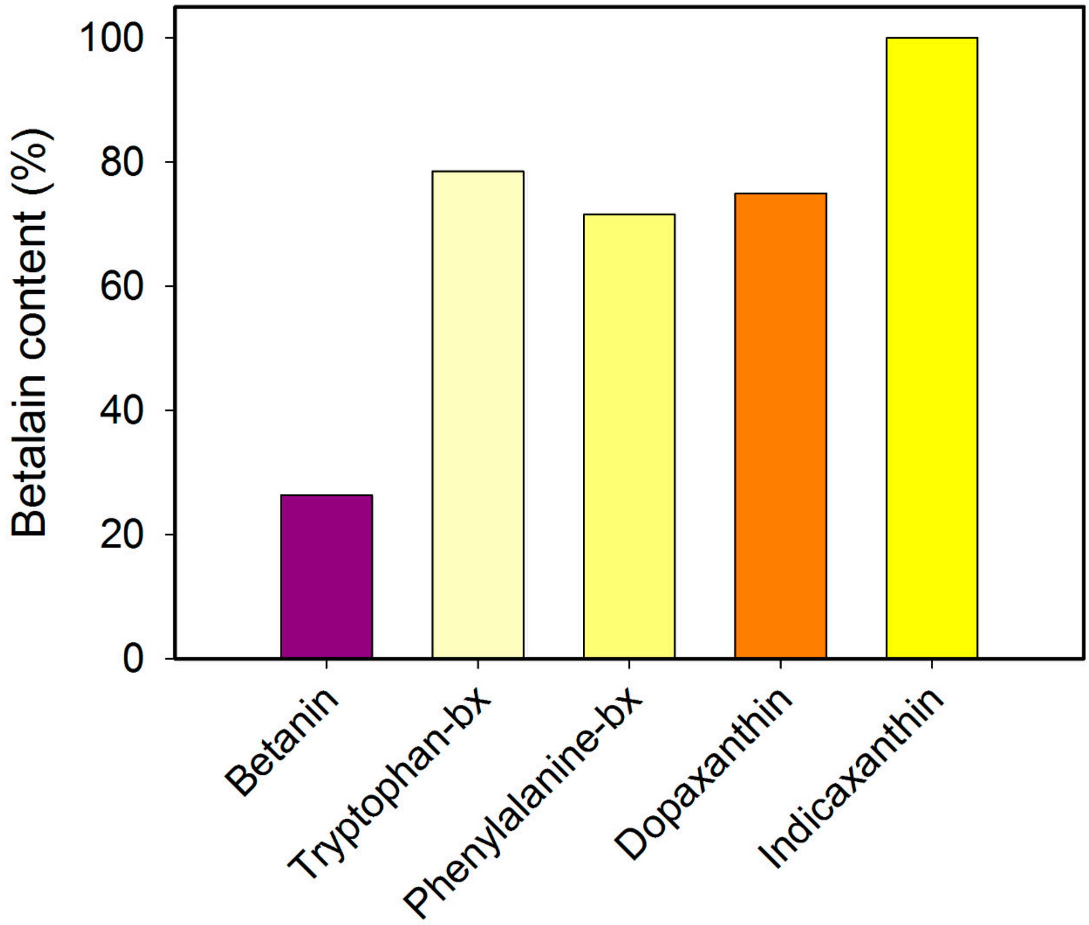

Supplementary Figure S2. Betalains stability in *C. elegans* S. medium after 48 hours at 20 ºC in the dark. The betalain content in the medium was obtained by measuring its absorbance spectrophotometrically at 536 nm for betanin and 480 nm for the betaxanthins (tryptophan-bx, phenylalanine-bx, dopaxanthin and indicaxanthin) at t=0 and t=48 h.

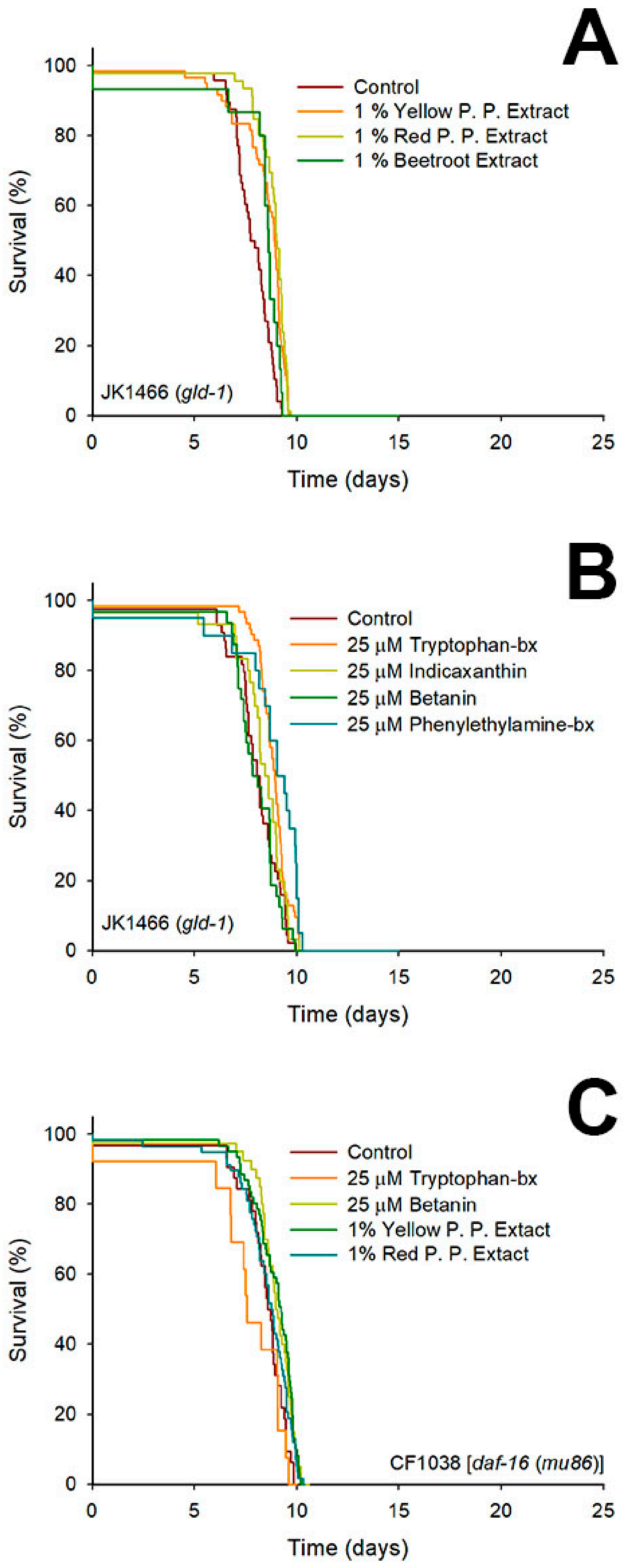

Supplementary Figure S3. Survival plots for *C. elegans gld-1* mutants treated with betalain-containing natural extracts and pure betalains. Natural extracts (**A**). Pure betalains (**B**). Survival plots for *C. elegans* CF 1038 [*daf-16(mu86)*] treated with tryptophan-betaxanthin, betanin and yellow and red prickly pears extracts (**C**).
